# Synthesis, Regulation and Degradation of Carotenoids Under Low Level UV-B Radiation in the Filamentous Cyanobacterium *Chlorogloeopsis fritschii* PCC 6912

**DOI:** 10.3389/fmicb.2020.00163

**Published:** 2020-02-12

**Authors:** Carole A. Llewellyn, Ruth L. Airs, Garry Farnham, Carolyn Greig

**Affiliations:** ^1^Department of Biosciences, College of Science, Swansea University, Swansea, United Kingdom; ^2^Plymouth Marine Laboratory, Plymouth, United Kingdom; ^3^Faculty of Medicine and Dentistry, University of Plymouth, Plymouth, United Kingdom

**Keywords:** cyanobacteria, carotenoids, UV, carotenogenesis, photoprotection, apocarotenoids, *Chlorogloeopsis fritschii*

## Abstract

Carotenoids in cyanobacteria play an important role in protecting against and in repairing damage against low level UV-B radiation. Here we use transcriptomics and metabolomic HPLC pigment analysis to compare carotenoid pathway regulation in the filamentous cyanobacterium *Chlorogloeopsis fritschii* PCC 6912 exposed to white light and to white light supplemented with low level UV-B. Under UV-B changes in carotenoid transcription regulation were found associated with carotenogenesis (carotenoid synthesis), photoprotection and carotenoid cleavage. Transcriptional regulation was reflected in corresponding pigment signatures. All carotenogenesis pathway genes from geranylgeranyl-diphosphate to lycopene were upregulated. There were significant increases in expression of gene homologs (*crtW*, *crtR*, *cruF*, and *cruG)* associated with routes to ketolation to produce significant increases in echinenone and canthaxanthin concentrations. There were gene homologs for four β-carotene-ketolases (*crtO* and *crtW*) present but only one *crtW* was upregulated. Putative genes encoding enzymes (CruF, CrtR, and CruG) for the conversion of γ-carotene to myxol 2′-methylpentoside were upregulated. The hydroxylation pathway to nostaxanthin via zeaxanthin and caloxanthin (gene homologs for CrtR and CrtG) were not upregulated, reflected in the unchanged corresponding pigment concentrations in zeaxanthin, caloxanthin and nostaxanthin, Transcripts for the non-photochemical quenching related Orange-Carotenoid-Protein (OCP) and associated Fluoresence-Recovery-Protein (FRP) associated with photoprotection were upregulated, and one carotenoid binding Helical-Carotenoid-Protein (HCP) gene homolog was downregulated. Multiple copies of genes encoding putative apocarotenoid related carotenoid oxygenases responsible for carotenoid cleavage were identified, including an upregulated apo-β-carotenal-oxygenase gene homologous to a retinal producing enzyme. Our study provides holistic insight into the photoregulatory processes that modulate the synthesis, photoprotection and cleavage of carotenoids in cyanobacterial cells exposed to low level UV-B. This is important to understanding how regulation of metabolism responds to a changing environment and how metabolism can be modulated for biotechnological purposes.

## Introduction

In contrast to the widely reported detrimental effects that UV has on cyanobacteria we know less about the effects of eustress or low level UV stress. In plants (and in animals) there is increasing evidence that eustress is good. Eustress confers a wide range of adjustment and adaption mechanisms to be invoked enabling and promoting survival (Jansen et al., 2008). Exposure to low levels of UV-B by cells actively promotes survival because it stimulates responses that help to protect against and repair UV-damage (Hideg et al., 2013).

Cyanobacteria are an ancient group of photosynthetic prokaryotes that have had an immense impact on the evolution of other organisms and on the global ecosystem. They are ubiquitous throughout the world and thrive in a wide range of marine and freshwater habitats as well as terrestrial and symbiotic habitats and make an important contribution to global primary production. The ability of cyanobacteria to survive under variable light environments and acclimate to a wide range of spectral conditions has contributed to their ecological and evolutionary success over millions of years (Rastogi et al., 2014; Gan and Bryant, 2015). Despite the importance of cyanobacteria in evolutionary and ecological understanding and more recently in biotechnology applications our understanding on cyanobacterial metabolism, particularly under low level eustress conditions is lacking.

Carotenoids are a group of tetraprenoid metabolites known to play an important role in protecting against photoooxidative damage (Liang et al., 2006). Terrestrial and freshwater cyanobacteria species typically produce a wider range of carotenoids than marine cyanobacteria and contain, in addition to the β-carotene and zeaxanthin found in marine species, keto-xanthophylls such as echinenone, canthaxanthin, and oscillaxanthin and myxol-glycosides (Jeffrey et al., 2011). Freshwater cyanobacteria can also contain the hydroxylated carotenoids caloxanthin and nostoxanthin (Schagerl and Donabaum, 2003), and the dicarboxylated synechoxanthin (Graham and Bryant, 2008).

Unlike in other photosynthetic cells where the majority of light for photosynthesis is harvested by carotenoids, in cyanobacteria (and in rhodophytes), light is additionally harvested for photosynthesis by phycobilin pigments within the phycobilisomes. Phycobilisomes are large light harvesting antenna protein complexes attached externally to the photosystem (Scholes et al., 2011) primarily associated with photosystem II (PSII), although they may also interact with PSI (Ashby and Mullineaux, 1999; Liu et al., 2013). Associated with phycobilisomes is a carotenoid binding protein, the Orange Carotenoid Protein (OCP). The OCP has a photoprotective quenching function protecting the reaction centers within PSI and PSII (Wilson et al., 2008). The protein consists of two domains, with a single keto-carotenoid molecule non-covalently bound between the two domains. OCP acts through non-photochemical quenching (NPQ) to very efficiently quench the excitation energy absorbed by the phycobilisomes (Boulay et al., 2008; Kirilovsky and Kerfeld, 2012; Bao et al., 2017b). This NPQ mechanism is the functional equivalent of the violaxanthin- zeaxanthin xanthophyll cycle present in chlorophytes and plants, and the diadinoxanthin –diatoxanthin xanthophyll cycle present in diatoms. The central carotenoid of the OCP requires a keto group for photoactivity, but the OCP can bind a diversity of these, e.g., the mono keto-carotenoid 3′-hydroxyechinenone, myxol 2′-methylpentosides, echinenone or canthaxanthin (Melnicki et al., 2016; Bao et al., 2017a). Our knowledge of the range of ketocarotenoids involved in this photoprotective mechanism is still emerging.

In addition to carotenoids associated with thylakoid membranes of PSI and PSII, in cyanobacteria, carotenoids can also be associated with cell envelope membranes. These carotenoids are different from, and less diverse than those associated with thylakoid membranes (Murata et al., 1981). Myxoxanthophyll, a monocyclic glycosidic carotenoid is associated with the cytoplasmic membrane and outer membrane of cyanobacteria, and is believed to play a role in the stabilization of cytoplasm and cell wall membranes (Graham and Bryant, 2009). Its glycoside attachment is typically a fucoside, rhamnoside or chinovoside (Takaichi and Mochimaru, 2007) but when the glycoside has not been characterized it is referred to as a myxol-2′-methylpentoside. Myxol-2′-methylpentoside can be both hydroxylated and ketolated to give 2-hydroxymyxol 2′-methylpentoside and 4-ketomyxol 2′-methylpentoside respectively.

Carotenogenesis, the synthesis of carotenoids, can be considered in five stages (Lohr, 2011). The first stage is to produce an isoprene building block unit consisting of isopentenyl diphosphate and its isomer dimethylallyl diphosphate. In cyanobacteria the steps to isoprene are undertaken solely using the methylerythritol phosphate (MEP) pathway (Pattanaik and Lindberg, 2015). The second stage is a stepwise condensation to produce phytoene. Phytoene represents the first committed point to carotenoid biosynthesis. The third stage is sequential desaturation and isomerization to produce lycopene. Lycopene represents the branch point to carotenoid diversity. The fourth stage is cyclization of the linear ends of lycopene to yield cyclic carotenes. The fifth stage is stepwise introduction of hydroxyl and keto groups to produce a diversity of xanthophylls (Figure 3). Although carotenogenesis in plants and, to some extent in green algae, has been well studied, it is only in the last decade or so that genes involved in carotenogenesis in cyanobacteria, algae and land plants have been identified (Chang et al., 2013).

Whilst most of the enzymes in carotenogenesis have been identified, less is known about carotenoid degradation. The first step in degradation involves enzymatic cleavage of the carotenoid by oxygenases to form a diverse range of bioactive apocarotenoids including volatiles, and photoprotective compounds (Kloer and Schulz, 2006). Carotenoid cleavage dioxygenases (CCDs) generate and modify these biologically important apocarotenoids by the oxidative cleavage of specific carotenoid double bond sites to form aldehyde or ketone products including retinal (Ahrazem et al., 2016). The roles that CCDs play in cyanobacteria are not fully understood.

These recent findings present opportunities to holistically better understand pathways to synthesis of keto-carotenoids, photoprotection and carotenoid turnover and degradation. In particular, the regulation of these carotenoid pathways in cyanobacteria under different conditions is relatively still poorly understood. Specifically, exposure to low level UV irradiation is of importance as it is a common feature of the habitat of many cyanobacterial groups. Thus studies on how carotenoids respond to low level UV-B stress on the cell and on the photosynthesis apparatus are required.

Cyanobacteria are highly diverse in their structure and development and have accordingly been classified into five subgroups or Sections (Castenholz, 2015). Most studies on carotenoids in cyanobacteria have been undertaken on the Section-I unicellular strains *Synechocystis* sp. PCC 6803 (Ruch et al., 2005; Vajravel et al., 2017) or *Synechococcus* sp. PCC 7002 (Albrecht et al., 2001; Graham and Bryant, 2008) with fewer studies on the Section-IV filamentous strains *Nostoc punctiforme* PCC 73102 and *Anabaena* sp. (also known as *Nostoc* sp.) PCC 7120 (Takaichi et al., 2005; Marasco et al., 2006). Here we investigate the carotenoid response to low level UV-B in the Section-V filamentous cyanobacterium *Chlorogloeopsis fritschii* PCC 6912. *C. fritschii* is a terrestrial species first isolated from the soils of a paddy field in India (Mitra, 1950). *C. fritschii* has been shown to be a robust thermophilic species with potential to produce a range of useful metabolites including carotenoids (Balasundaram et al., 2012; Kultschar et al., 2019). *C. fritschii* was the first freshwater cyanobacterial species for which detailed compositional analysis of carotenoids was determined (Evans and Britton, 1983). More recently there has been renewed interest in *C. fritschii* for its ability to adapt to the far red light (Airs et al., 2014; Ho and Bryant, 2019). Transcription and pigment metabolite profiling were undertaken on cultures exposed to white light and compared to those exposed to white light supplemented with low level UV-B.

## Materials and Methods

### Experimental Conditions

*Chlorogloeopsis fritschii* (Mitra) PCC 6912 was inoculated at 1:50 dilution from a master culture and cultivated in 5 L Erlenmeyer flasks containing 2 L BG11 media with 10 mM HEPES buffer at pH 7.5. The culture was perfused with 1% CO_2_ and was maintained at 38°C under constant white light (410–750 nm: Grolux fluorescent tubes) of 60 μmol photons m^–2^ s^–1^. At exponential growth phase, cells were harvested and transferred to nine 500 mL quartz Erlenmeyer flasks containing 200 mL of fresh BG11, and 10 mM HEPES at pH 7.5 to give a concentration of 0.44 g L^–1^ wet weight (approximating to 0.04 g L^–1^ dry weight). All 9 cultures were exposed to constant white light (410–750 nm: Grolux fluorescent tubes) of 60 μmol m^–2^ s^–1^ photons for 4 days and 4 h (100 h in total). Three flasks were exposed to white light with no UV-B supplementation acting as the control (white), three flasks were exposed to white light supplemented with UV-B for the final 4 h of the experiment (4h) and, three flasks were exposed to white light supplemented with 4 h of UV-B radiation each day for 4 days (4h4d). Flasks exposed to UV-B were placed 10 cm from UV-B tubes supplying 3 μmol m^–2^ s^–1^ at wavelength range 300–310 nm. The experiment was set up, guided using prior experiments, where photosynthetic efficiency (Fv/Fm) as previously described (Steele et al., 2018), was measured so that the level of UV-B exposure did not impact detrimentally on photosynthetic efficiency (Supplementary Figure S1). At the end of the experiment samples all 9 flask were placed on ice and were centrifuged (3000 *g*) at 4°C. The pelleted biomass was snap frozen in liquid nitrogen before storage at −80°C. Transcriptomics was undertaken on the white light (control) and 4h4d samples. Targeted metabolomics to determine pigments was undertaken on the white, 4h and 4h4d samples.

### RNA Preparation and Sequencing

For the white light control and the 4h4d samples, RNA was extracted with Trizol followed by terminator exonuclease digestion to enrich for mRNA and subsequently cleaned using a Qiagen RNeasy column. RNA sequencing was conducted at the Centre for Genomic Research, Institute of Integrative Biology at the University of Liverpool, United Kingdom, L69 7ZB, using the Life Technologies SOLiD sequencing platform. For each sample, at least 49,034,856 sequences were obtained (50 bp, min average quality 20 as per manufacturer specifications; per sample average sequence number: 57,516,996.44).

### Bioinformatic Analysis

Alignment of reads was carried out using the *C. fritschii* PCC 6912 genome as reference. The sequences obtained for each sample were aligned on to the reference using bowtie version 0.12.7, using the color space option. Prior the alignment step the sequences required the conversion to a pseudo-fastq file required as input for bowtie. For each of sequence, only the best alignment was reported by bowtie, or one was randomly chosen if many were equally best. The average percentage of unambiguously aligned sequence was 47.67%, with a minimum of 35.9% and the maximum equal to 51.9. Considering the known 6,968 genes, an average of 83.11% of these were identified as expressed across all the samples (ranging between 73.95 and 87.15%). All the obtained alignment files were processed using HTSeq-count (Anders et al., 2015), and reads aligning to the reference genome sequences were counted according to the gene features that they mapped to, as defined in the GTF files.

The differential expression analyses between white and the 4h4d samples were performed using R (version 2.14) and edgeR package. The gene-counts were normalized using “loess smooth” method form the ‘limma’ package. The “GLM” model was applied to the normalized data (with edgeR package) and the dispersion related to each gene (genewise dispersion) and the pairwise group comparisons were performed to identify differentially expressed genes for each of the three possible group comparisons. For each contrast, each gene with a *p*-value below 0.05 (after adjusting for multiple testing effect using the False Discovery Rate approach, Benjamini and Hochberg, 1995) were selected as differentially expressed for that contrast. Significant changes in regulation were defined as log2 fold change ≤ −1.5 or ≥1.5 and *p*-adj < 0.05. Data produced in this study can be found within an NCBI BioProject with accession number PRJNA545395.

### Pigments

For white, 4h and 4h4d samples, cell pellets (80–170 mg wet weight) were transferred to extraction tubes containing 1 mm glass beads and extracted into 100% methanol using an ultrasonic probe (35 s; 50 W), and 1 mm glass beads. Pellets were repeat extracted with 1 ml methanol five times. Extracts were clarified and analyzed according to the method of Zapata et al. (2000) by injecting onto a Waters Symmetry C8 reverse phase column (150 × 4.6 mm, 3.5 μm particle size) on a Thermo Accela Series HPLC system with chilled autosampler (4°C) and photodiode array detector. The HPLC was calibrated using a suite of standards purchased from DHI (Denmark). The standards were used to generate response factors used for quantification. For pigments where standards were not available (caloxanthin and nostoxanthin) the response factors of the most closely related standard, zeaxanthin, was used. Pigments were identified based on retention time, spectral match using photo-diode array and LC/MS (Supplementary Table S1). Probability (p) of significance between white light control and the 4h4d samples was determined using a Student’s *t*-test test with a two tailed distribution.

## Results

**FIGURE 1:**
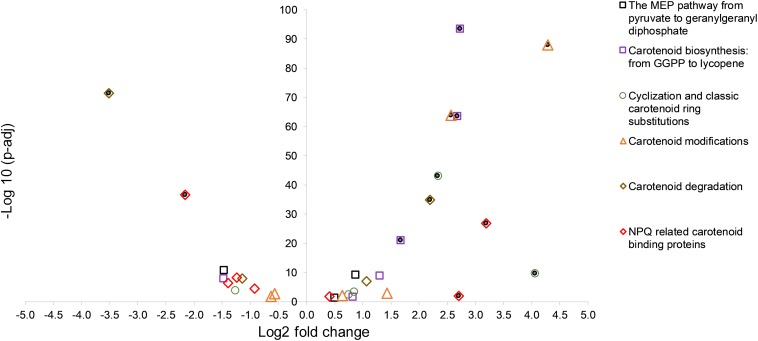
A volcano plot showing significance versus log fold change in expression of carotenoid synthesis and metabolism in *C. fritschii* PCC 6912 in response to supplementary low level UV-B (4h4d samples). Filled circles indicate significance with −1.5 > log2 fold change > 1.5 *p*.adj < 0.05.

An overview of the log2 fold changes of differential transcriptional expression in carotenoid and carotenoid related gene homologs in *C. fritschii* PCC 6912 when exposed to supplementary low level UV-B (4h4d) is shown in Figure 1. Further details including levels of expression can be found in Supplementary Table S2. Statistically significant differential transcription regulation changes across carotenogenesis, photoprotection and carotenoid cleavage were observed (Figure 1).

### Carotenogenesis

Focusing on the early stages of biosynthesis of the carotenoids, none of the gene homologs encoding enzymes up to the point of geranylgeranyl diphosphate were differentially upregulated and one putative kinase was slightly differentially downregulated (EC:2.7.1.148: WP_016876009.1: log2 fold change −1.47) in response to the UV treatment (Supplementary Figure S3).

**FIGURE 2:**
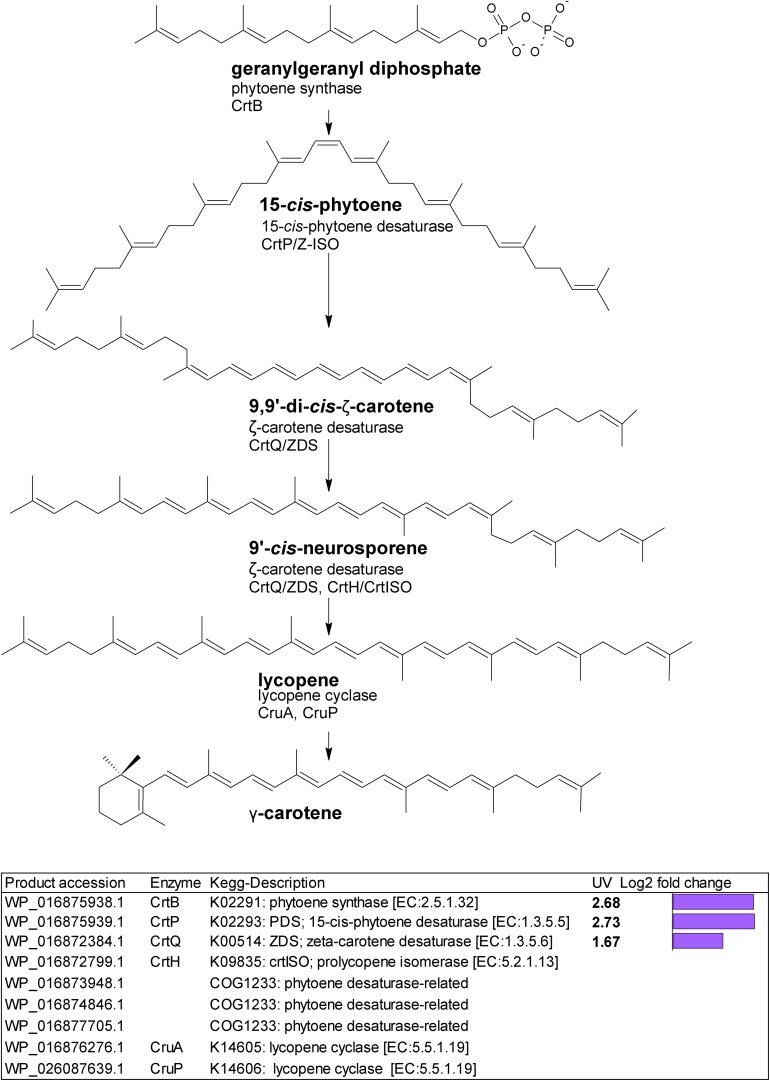
Biosynthetic pathway from geranylgeranyl diphosphate to lycopene. Genes identified by orthology for enzymes which can catalyze pathway steps are shown together with significant changes in regulation (with –1.5 > log2 fold change > 1.5 and *p*-adj < 0.05) under UV-B treatment (4h4d samples) illustrated by the purple bars.

Gene homologs encoding enzymes which catalyze subsequent steps leading to lycopene were differentially upregulated (Figure 2). These were a putative *crtB* phytoene synthase (WP_016875938.1) with a log2 fold change of 2.68; a putative *crtP*; 15-*cis*-phytoene desaturase (WP_016875939.1) with a log2 fold change of 2.73, and a putative *crtQ*; ζ-carotene desaturase (WP_016872384.1) with a log2 fold change of 1.67. We also found three putative COG1233 phytoene desaturase-related gene homologs (Figure 2).

Lycopene represents a crucial step in carotenoid synthesis, being the precursor to the branch point which generates carotenoid diversity (Lohr, 2011). In cyanobacteria, lycopene is the precursor to γ- and β-carotene. Gene homologs encoding two lycopene cyclases, CruA and CruP which catalyze cyclization from lycopene to produce γ- (one cyclic ring) and β-carotene (two cyclic rings) were not differentially regulated under UV.

**FIGURE 3:**
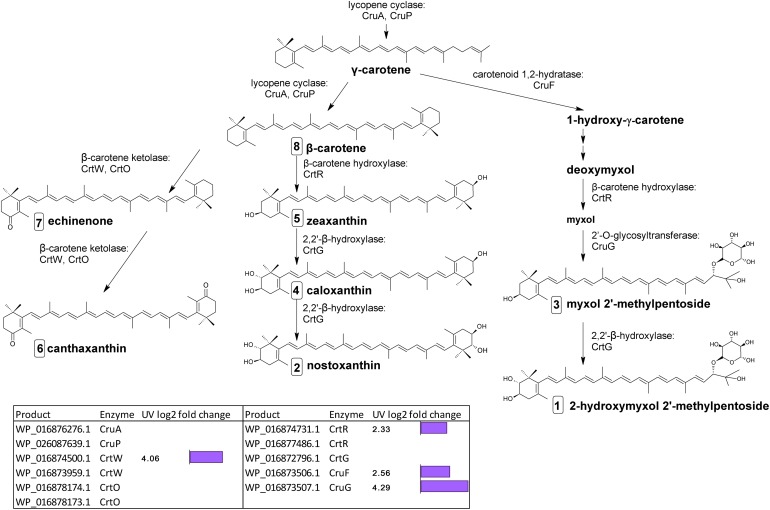
Biosynthetic pathway of carotenoids from γ-carotene in *C. fritschii* PCC 6912. Genes identified by orthology for enzymes which can catalyze pathway steps are shown together with significant changes in regulation (with –1.5 > log2 fold change > 1.5 and *p*-adj < 0.05) under UV-B treatment (4h4d samples) illustrated by the purple bars. Numbers in squares indicate the order of elution on the HPLC corresponding to Figure 4. For myxol 2′-methylpentoside and 2-hydroxymyxol 2′-methylpentoside the glycoside is represented as a fucoside.

The final stages of biosynthesis from γ- and β-carotene leading to the diversity of carotenoids found in *C. fritschii* involves the stepwise introduction of ketol, hydroxyl, or glycosyl groups using ring-modifying ketolase, hydratase, hydroxylase, and transferase enzymes (Figure 3). Some of these enzymes are promiscuous for carotenoid substrates (Figure 3).

From β-carotene, ketolisation produces echinenone and canthaxanthin. Ketolisation also converts myxol-2′-methylpentoside to 4-ketomyxol 2′-methylpentoside. However, notably we did not observe any 4-ketomyxol 2′-methylpentoside in our HPLC chromatograms (see section Carotenoid Metabolites). We found four β-ketolase homologs involved in catalyzing the conversion of β-carotene to echinenone and canthaxanthin; two of the CrtO type and two of the CrtW type. Expression of one homolog of each type was not detected in this study, and the expressed *crtO*, WP_016878174.1 was slightly but not significantly downregulated under UV (log2 fold change −1.27). However, the expressed gene encoding a CrtW ketolase homolog (WP_016874500.1) was highly differentially upregulated under UV with a log2 fold change of 4.06 (Figure 3). β-carotene can also be converted by hydroxylation to zeaxanthin, caloxanthin and nostoxanthin (Figure 3). This uses a β-carotene hydroxylase and a 2,2′-β-hydroxylase. There are two genes encoding putative CrtR type beta-carotene hydroxylases, a major (WP_016874731.1) and a minor (WP_016877486.1) according to transcription levels. In this pathway, only the major *crtR* homolog was upregulated under UV-B (WP_016874731.1) with a log2 fold change of 2.33.

In *C. fritschii* a number of gene homologs encode enzymes (CruF, CrtR, CruG) to convert γ-carotene to myxol glycosides to produce myxol-2′-methylpentoside (Graham and Bryant, 2009). We found upregulation of all these genes under UV indicating an activation of this pathway (Figure 3). We observed the largest upregulation for a glycosyltransferase *cruG* (WP_016873507.1: log2 fold 4.29). CruG is required for the conversion of myxol to the myxol 2′-methylpentoside.

We also identified a *crtG* homolog (WP_016872796.1) encoding for the hydroxylation of zeaxanthin to caloxanthin and nostoxanthin and of myxol 2′-methylpentoside to 2-hydroxymyxol 2′-pentoside. *CrtG* was not found to be differentially regulated under UV-B.

In terms of the regulation of carotenoid biosynthesis, the global transcription factor NtcA reported to regulate carotenoid biosynthesis in *Nostoc* PCC 7120 (Sandmann et al., 2016) was not upregulated under UV in *C. fritschii* PCC 6912. However, we did observe the presence of signature NtcA binding sites in *C. fritschii* PCC 6912 promoter regions of the *crtPB* operon, *crtO* and *crtW* (Supplementary File S1), which indicates a functional role in the NtcA control of carotenoid transcription in *C. fritschii* PCC 6912, likely to be modulated by binding of cofactors such as 2-oxoglutarate (Tanigawa et al., 2002).

### Photoprotection

Next, we looked for carotenoid binding proteins related transcripts known to be involved in photoprotection. We found a number of orange carotenoid protein (OCP) related transcripts involved in photoprotection of the phycobilisome (PBS) and these showed changes in regulation under UV. One gene homolog encoding a full OCP1 (WP_016875600.1) was found situated next to the gene homolog encoding fluorescence recovery protein (WP_016875601.1); both of these were strongly upregulated in UV (log2 fold change 3.19 and 2.71 respectively) (Table 1).

**TABLE 1:** Expression changes in OCP and CCD related gene homologs.

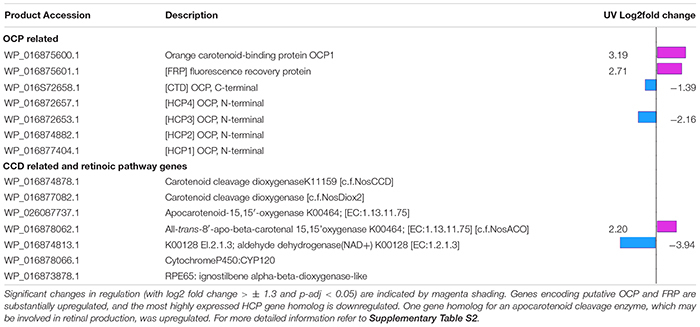

Gene homologs encoding N- and C-terminal O, a helical carotenoid protein (HCP) and a C-terminal domain (CTD respectively, were also found. There were four HCPs and one CTD paralog (WP_016872658.1), as reported in *Anabaena* sp. PCC 7120 (Lopez-Igual et al., 2016). We identified gene homologs for HCP1 (WP_016877404.1), HCP2 (WP_016874882.1), HCP3 (WP_016872653.1) and HCP4 (WP_016872657.1), which is adjacent to the CTD gene homolog. The gene homolog for CTD (WP_016872658.1) and for one HCP (WP_016872653.1) were downregulated under UV (log2 fold changes −1.39 and −2.16 respectively (Table 1).

### Carotenoid Cleavage Enzymes

Finally, we looked for gene homologs associated with enzymes responsible for the cleavage of carotenoids. We identified five carotenoid cleavage dioxygenase encoding gene homologues (CCDs); these were associated with the retinal pigment epithelial membrane protein family [COG3670 enzymes (Table 1 and Supplementary Table S2). We compared protein sequences to those of the three genes defined in *Nostoc* sp. PCC 7120 (Marasco et al., 2006) by multiple sequence alignment. We found WP_016874878.1 was orthologous to NosCCD (all1106), which cleaves C9–C10 double bonds (Scherzinger and Al-Babili, 2008), and WP_016877082.1 to NosDiox2 (all4895) which cleaves apocarotenoids at C13–C14, C13′–C14′, and C15–C15′ double bonds (Heo et al., 2013).

WP_016878062.1 and WP_026087737.1 had protein sequence homology to NosACO (all4284), which cleaves monocyclic or acyclic carotenoids at C15–C15′ double bonds to generate retinal (Ruch et al., 2005; Scherzinger et al., 2006). The fifth gene WP_016873878.1 was for a much longer RPE65 with homology to the lignostilbene-alpha, beta-dioxygenase protein in *Nostocales*, and unlike the other enzymes identified, this protein contains only two of the four conserved active site histidines characterizing CCD enzymes (Marasco et al., 2006). Only WP_016878062.1 generating retinal showed a change in transcriptional expression in UV light (log2 fold 2.20).

*C. fritschii* PCC 6912 is one of only 65 cyanobacterial species to possess orthologs for genes for all three enzymes in the putative retinoic pathway (Miles et al., 2019). These are the UV upregulated gene homolog for carotenoid oxygenase (WP_016878062.1), to act on β- carotene to produce retinal, a transcript encoding an aldehyde dehydrogenase (WP_016874813.1 6) to produce retinoic acid from this, which was significantly downregulated under UV (log2 fold −3.94), and an ortholog to the gene for Cyp120 (WP_016874813.1 6) to produce 4-hydroxy-retanoinc acid for which expression was not observed (Table 1 and Supplementary Table S2).

### Carotenoid Metabolites

Chlorophyll *a* concentrations, consistent with the intentional low dose of UV-B, were not significantly different across the nine samples [average 865 ± 128 μg g^–1^ wet weight (969 nmol g^–1^ ± 143): *p* = 0.88 for white and 4h4d]. The total carotenoid pool equated to approximately 28% of that of chl-*a* (on a molar basis). Likewise the carotenoid pool was determined not to be significantly different between the 9 samples [154 ± 12 μg g^–1^ (276 nmol g^–1^ ± 38: *p* = 0.18 for white and 4h4d)].

**FIGURE 4:**
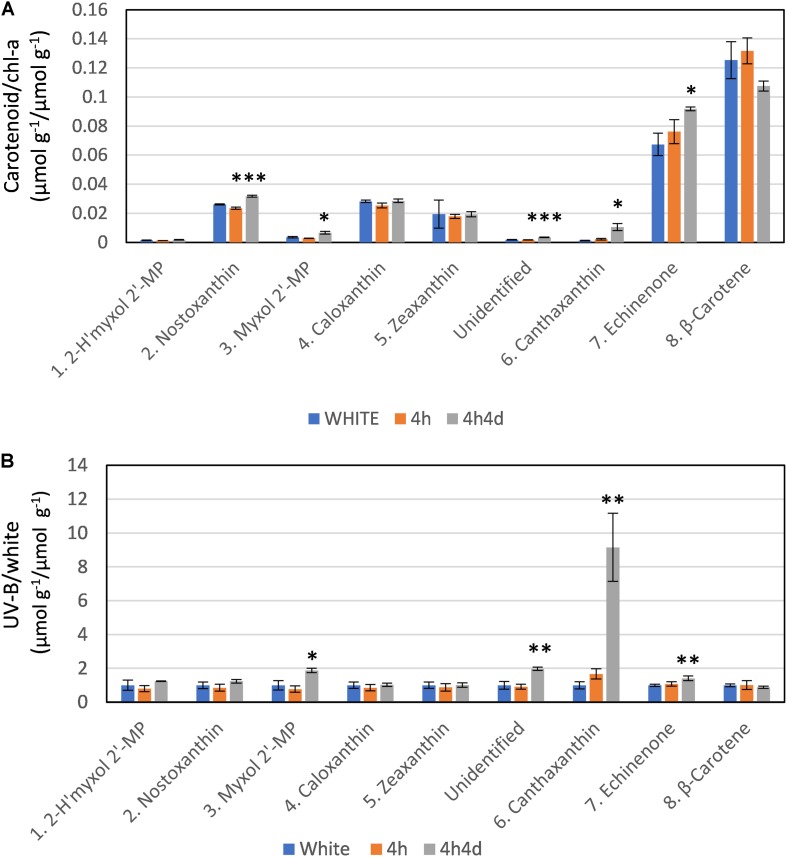
Response of carotenoids to white light and white light supplemented with low level UV-B exposure (4h and 4h4d samples). Carotenoids shown in order of HPLC eluted retention time from polar to non-polar. For HPLC retention time and UV-Vis spectral and LC/MS characterization refer to Supplementary Figure S2 and Supplementary Table S1. **(A)** Concentration (micromoles per gram of dry weight) of the carotenoid as a ratio of chlorophyll-a concentration (micromoles per gram of dry weight) under the three conditions. **(B)** Concentration of pigments (micromoles per gram of dry weight) in UV-B exposed samples (4h and 4h4d) as a ratio to the concentration (micromoles per gram of dry weight) in white light. 2- h′myxol 2′-MP, 2-hydroxymyxol 2′-methylpentoside (tentative identification) and myxol 2′-MP, myxol 2′-methylpentoside. Significance difference between white light and 4h4d samples: ^∗^ = 0.05 > *p* ≥ 0.01, ^∗∗^ = 0.01 > *p* ≥ 0.001, and ^∗∗∗^ = *p* < 0.001.

The changes observed in levels of carotenoids under UV-B were consistent with the changes observed in transcriptome regulation. The main carotenoids identified in both white and UV supplemented samples were, in order of HPLC elution from polar to non-polar, 2-hydroxymyxol 2′-methylpentoside (tentative), nostoxanthin, myxol 2′-methylpentoside, caloxanthin, zeaxanthin, canthaxanthin, echinenone and β-carotene (Supplementary Figure S2 and Supplementary Table S2). Notably, we did not detect any 4-ketomyxol 2′methylpentoside. β-carotene and echinenone were the two most abundant carotenoids that we detected (Figure 4A). Across all samples these two least polar of the carotenoids were at least double the concentration of other carotenoids (Figure 4A). We found echinenone and β-carotene to increase after 4 h suggesting that the PSI enriched pigments increase whereas the myxol-fucosides, zeaxanthin possibly more associated with PSII decreased in the first 4 h. After 4h4d these more polar carotenoids showed increases whereas echinenone and β-carotene each show an opposite response with echinenone continuing to increase and β-carotene decreasing (Figure 4A).

Whilst there were some declines in carotenoid concentrations relative to chl-*a* after 4h of the UV treatment, notably for the more polar carotenoids (Figure 4), we focus here, in correspondence with transcriptome data, on changes observed after 4h4d of UV. For the 4h4d UV samples carotenoids either had similar or increased concentration compared to the white light samples. The only exception was for β-carotene which showed a decrease, although statistically this was not significant (*p* = 0.14). Three carotenoids showed significant increases measured in μg g^–1^ wet weight. Echinenone showed a significant increase (*p* = 0.019) from 36 ± 6 μg g^–1^ (65 nmol g^–1^) under white light to 50 ± 24 μg g^–1^ (92 nmol g^–1^) under the 4h4d UV exposure conditions. Myxol 2′-methylpentoside and canthaxanthin, whilst both present at relatively low concentration, showed significant (*p* = 0.016 and 0.019 respectively) increases after 4h4d of UV-B exposure increasing from a mean of 2.5 μg g^–1^ (3.5 nmol g^–1^) to 4.8 μg g^–1^ (6.5 nmol g^–1^) and from 0.6 μg g^–1^ (1.1 nmol g^–1^) to 5.9 μg g^–1^ (10 nmol g^–1^) respectively. The most notable difference was for canthaxanthin which showed a nine fold increase in the 4h4d samples when exposed to UV-B (Figure 4B).

## Discussion

### Photosystem Complexes

Early work found carotenoids distributed differently between the two photosystem complexes (Evans and Britton, 1983): Photosystem I (PSI) of *Chlorogloeopsis* was found to be preferentially enriched in β-carotene and echinenone whereas PSII was enriched in the more polar carotenoids particularly myxoxanthophyll and zeaxanthin: although notably the location of the majority of echinenone could not be assigned (Evans and Britton, 1983). Association of echinenone with PSI was confirmed more recently in *Synechocystis* sp. PCC6803 (Vajravel et al., 2017). The increases we observed after 4 h in echinenone and β-carotene suggests that the PSI enriched pigments increase whereas the myxol 2′-methylpentosides and zeaxanthin possibly more associated with PSII decrease. Photosystem II (PSII) is the most light-sensitive complex in the photosynthetic apparatus. Strong light inhibits (photoinhibition) of the activity of PSII. Recent findings suggest that environmental stressors act primarily by inhibiting repair of PSII (Murata et al., 2007). Our results suggest that initially UV-B exposure causes a reduction in carotenoids whereas after 4h4d of exposure, PSII is recovered. This recovery could be activated by the observed increased levels of canthaxanthin.

### Carotenoid Transcription Regulation

We found three major types of transcriptional change under UV-B associated with carotenoids; firstly, that associated with carotenoid synthesis (carotenogenesis); secondly photoprotecting-NPQ related, and thirdly, associated with carotenoid cleavage.

### Carotenogenesis

The first stage of carotenoid production produces active isoprene; in cyanobacteria this is undertaken solely using the MEP pathway (Lohr et al., 2005; Paniagua-Michel et al., 2012). This is converted into geranylgeranyl diphosphate, the substrate for phytoene synthase. These initial terpenoid biosynthesis steps were not affected by UV-B (Supplementary Figure S3).

Phytoene synthase (CrtB) performs the rate-limiting entry step into carotenoid biosynthesis (Paniagua-Michel et al., 2012), and this together with phytoene desaturase (CrtP), and ζ-carotene desaturase CrtQ, transcripts for all of which we found upregulated under UV, provides the pathway to lycopene (Figure 2). We also observed three putative COG1233 phytoene desaturase-related homologs requiring further investigation. Lycopene represents a crucial step in carotenoid synthesis, generating carotenoid diversity. Upregulation of this pathway opens the gateway to higher carotenoid production, supplying the precursors needed to enable increased photoprotection. It is notable that the cyclases needed to convert lycopene to the diverse carotenoids seen were not transcriptionally upregulated; the pool of enzymes present may be sufficient to allow increased lycopene conversion.

Extending the carotenoid pathway to ketocarotenoids is achieved via carotenoid ketolases; however, these differ in their catalytic properties in different species. For example CrtO in *Nostoc* PCC 73102 is a diketolase rather than a monoketolase as in *Synechocystis* PCC 6803 (Schopf et al., 2013). In *Anabaena* sp. PCC 7120 CrtO and CrtW were functionally characterized to be associated with the conversion of β-carotene to echinenone and canthaxanthin and myxol to 4-keto-myxol respectively (Mochimaru et al., 2005; Takaichi and Mochimaru, 2007). *Nostoc* PCC 73102 has two carotenoid CrtW ketolases with different properties (CrtW148 and CrtW38) (Steiger and Sandmann, 2004; Takaichi and Mochimaru, 2007; Makino et al., 2008). Enhanced production of canthaxanthin in high light stress in *N. punctiforme* PCC 73102 has been attributed to the specific upregulation of *crtW148* (along with *crtB*), when it is thought to take over from the other ketolases (Schopf et al., 2013). This contrasts to the light induced upregulation of both *crtW* and *crtO* found in *Nostoc* PCC 7120 (Sandmann et al., 2016).

In *C. fritschii* PCC 6912 there are two genes encoding putative CrtO ketolases and two genes were also identified encoding putative CrtW ketolases. Only one of these four ketolase gene homologs, *crtW* (WP_016874500.1), was transcriptionally upregulated under UV with a log2 fold change of 4.06 (Figure 3). Protein sequences for WP_016874500.1 and *Nostoc* PCC 73102 CrtW148 have a 68.46% identity, and the second *crtW* (WP_016873959.1) encodes a protein with a 69.29% identity to *Nostoc* PCC 73102 CrtW38. The pattern of transcription in *C. fritschii* indicates that upregulation of the CrtW148 -like WP_016874500.1 observed may be the sole transcriptional change responsible for the increase in keto-carotenoids present after UV exposure.

The transcription data indicates an increase in carotenoid synthesis, and a shift toward the production of the ketolated carotenoids echinenone and canthaxanthin under low UV. This was reflected in the significant increases we observed in concentrations of canthaxanthin and echinenone in the HPLC chromatograms. Homologs for all genes (*cruF, crtR, cruG*) known to contribute to the myxol biosynthesis pathway were upregulated. But notably we did not observe any of the ketolated 4-ketomyxol 2′-methylpentoside in our chromatograms. Echinenone is known to play a key role as the core carotenoid to OCP and red carotenoid protein (RCP), and myxol glycosides are known to provide cell wall photoprotection. However, the role of these carotenoids in photoprotection is still unclear and recent evidence suggests that a wider range of carotenoids including myxol 2′-methylpentosides and canthaxanthin may also play a role in OCP and RCP (Melnicki et al., 2016). In contrast, the hydroxylation pathway to produce zeaxanthin, caloxanthin and nostoxanthin was not affected by UV and again this was reflected in the levels of these pigments which were largely unaltered under UV.

The global transcription factor NtcA regulates carotenoid biosynthesis in *Nostoc* PCC 7120 through 2-oxoglutarate mediated binding (Sandmann et al., 2016). In *C. fritschii* PCC 6912 this is encoded by WP_016874320.1, which was not upregulated under UV. Regulation through NtcA via signaling molecules rather than by its increased transcription enables modulation in response to the C/N balance and cellular redox state, and is implicated in regulation in response to high light in *Nostoc* PCC 7120 (Sandmann et al., 2016). Promotor analysis of *C. fritschii* PCC 6912 carotenoid pathway gene homologs in comparison to *Nostoc* PCC 7120 sequences revealed the presence of signature NtcA binding sites giving evidence for similar NtcA regulation of carotenoid biosynthesis in *C. fritschii* PCC 6912 (For detailed analysis refer to Supplementary File S1).

### Photoprotection

We found substantial upregulation of putative genes encoding the NPQ related water soluble photoactive proteins OCP and Fluorescence Recovery Protein (FRP). These proteins together provide the main mechanism by which the PBS is protected from excess light using non-photochemical quenching. The role of Helical Carotenoid Protein (HCP) is still emerging (Bao et al., 2017a). *C. fritschii* PCC 6912 has a complement of genes analogous to that found in *Anabaena*: i.e., HCP1-4 and one CTD paralog. In *Anabaena*, HCP4 containing canthaxanthin bound the PBSs, enabling constitutive fluorescence quenching, and HCP2 and HCP3 exhibited strong singlet oxygen quenching activity, while HCP1 is thought to have an alternate role in stress protection (Lopez-Igual et al., 2016).

We found a putative gene encoding a HCP3, situated nearby the HCP4-CTD gene homologs, and the CTD were downregulated under the UV treatment: Expression of the other HCP transcripts was unchanged. This indicates that, rather than providing additional UV stress protection, HCPs may have alternate roles in *C. fritschii*. The downregulation of HCP could be interpreted as a requirement to free up bound keto-carotenoids to increase the efficacy of OCP function. The roles of the HCP proteins require further investigation.

### Carotenoid Cleavage

Carotenoids are cleaved to form a number of products including retinal. Cyanobacterial genomes have 1-5 CCD gene homologs, with higher numbers in the filamentous cyanobacteria (Cui et al., 2012). *Nostoc* sp. PCC 7120 has three enzymes, exhibiting different substrate preferences (Marasco et al., 2006). *C. fritschii* has four corresponding CCD gene homologs including two apocarotenoid oxygenases. Only one of these, which may be involved with retinal synthesis, was upregulated in UV. In cyanobacteria, apocarotenoids act as photoprotective pigments in thylakoid membranes, and retinal acts as the chromophore for rhodopsins, thought to act as photosensory receptors (Jung et al., 2003). However, expression of the *C. fritschii* rhodopsin (WP_016877934.1) was not modulated by UV light. Cleavage products may also be involved in carotenoid turnover or be involved in signaling or regulation. Induction of an apocarotenoid modulating enzymes has been suggested to allow scavenging of apocarotenoids produced by photodestruction in high light (Ruch et al., 2005). Along with other apocarotenoids, retinal is an industrially valuable metabolite. A greater understanding of the function and regulation of these genes provides useful insights for increasing yields of apocarotenoids including the retinals in industrial biotechnology.

### Carotenoid Concentrations

β-carotene and echinenone, the most lipophilic carotenoids we detected, were present in the highest concentrations under white and UV supplemented conditions. Both these pigments act as precursors to canthaxanthin. As a keto-carotenoid canthaxanthin is known to retard the formation of hydroperoxide more efficiently and be more effective as an antioxidant than carotenoids without a keto group (Terao, 1989). Whilst canthaxanthin was present at much lower concentrations under visible and UV, under UV canthaxanthin showed by far the largest change with a ninefold increase in 4h4d exposure. Canthaxanthin has previously been shown to be preferentially formed over zeaxanthin in cells exposed to strong light (500 or 1200 μmol m^–2^ s^–1^) with UV-B radiation and, to be a better protectant against solar radiation (Albrecht et al., 2001). An increase in canthaxanthin and a decrease in β-carotene under UV exposure has also been reported in terrestrial cyanobacteria (Lakatos et al., 2001). Also the increases in myxol 2′-methylpentoside and echinenone we observed in the 4h4d UV exposed samples are consistent with other studies where secondary carotenoids have been shown to increase under UV (Ehling-Schulz et al., 1997; Ivanov et al., 2000).

Overall, the changes we found in the carotenoid metabolites in UV exposed cells were consistent with the changes observed in the differential transcription levels, providing strong evidence for pathway activation. The significant increases in both myxol 2′-methylpentoside and canthaxanthin under UV-B were consistent with the significant increases in expression of gene homologs associated with *crtW*, *crtR*, *cruF*, and *cruG*. However it should be noted that because some genes are involved in multiple reactions and in some cases there are multiple copies then not all genes could be definitively assigned to the production of particular carotenoids.

## Conclusion

New insight is provided into the regulatory processes that modulate the synthesis, photoprotection and degradation of carotenoids in the filamentous species of cyanobacteria *C. fritschii* under low UV-B. Through differential transcript analysis we demonstrated a UV-B induced upregulation of the pathway encoding the initial stages of carotenoid synthesis, producing phytoene and lycopene, but not the cyclases producing β-carotene. Gene homologs encoding enzymes responsible for the synthesis of the keto-carotenoids echinenone and canthaxanthin and of myxol 2′-methylpentoside were upregulated. Metabolite analysis confirmed increases in the keto-carotenoids echinenone and canthaxanthin and of myxol 2′-methylpentoside. This may indicate use of a relatively large β-carotene pool as a mechanism for cellular response to UV via conversion to canthaxanthin via echinenone, with more gradual replacement by lycopene cyclases.

Understanding gene regulation enables optimization of the photochemical performance of cyanobacteria to improve yields of specific carotenoids, such as keto-carotenoids and other valuable biotechnology metabolites such as the apocarotenoid, retinal. Thus our study is relevant to understanding the ecological survival of cyanobacteria under changing climatic conditions and to a society increasingly interested in using cyanobacteria for the production of useful metabolites.

## Data Availability Statement

The datasets generated for this study can be found within an NCBI BioProject with accession number PRJNA545395 (data to be released on publication).

## Author Contributions

CL secured the funding, conceived the ideas, oversaw the work, and wrote the manuscript. GF secured funding from NBAF and undertook the experiments. CG undertook bioinformatics and contributed to writing the manuscript. RA undertook HPLC of pigment samples, provided the data, and commented on the manuscript.

## Conflict of Interest

The authors declare that the research was conducted in the absence of any commercial or financial relationships that could be construed as a potential conflict of interest.

## Supplementary Material

FIGURE S1 Prior experiment to determine level of UV-B at wavelength range 300–310 nm exposure that would not impact detrimentally on photosynthetic efficiency (Fv/Fm). White light, UV 0, 10, and 20 cm represent the control and the distance of the flasks containing *C. fritschii* PCC 6912 from the UVB source. Average of three replicates with standard deviation. From this, for the main experiment, flasks were placed 10 cm from UV-B tubes measured as supplying 3 μmol m^–2^ s^–1^.

FIGURE S2 HPLC chromatogram of *C. fritschii* PCC 6912.

FIGURE S3 Gene regulation of terpenoid biosynthesis via the MEP/DOXP pathway to geranylgeranyl diphosphate in *C. fritschii* WP_016876009.1 was down-regulated (blue shading), other genes were identified but transcription was not observed (light green) or were not differentially regulated (dark green shading).

TABLE S1 Retention time and UV/vis absorption maxima of main peaks detected during HPLC of *C. fritschii* PCC 6912.

TABLE S2 *C. fritschii* carotenoid related gene expression in UV supplemented white light.

FILE S1 Promotor signatures of potential NtcA regulation of carotenoid transcription in *C. fritschii* PCC 6912.
