# Supplementary figures and images for: Synthesis, Regulation and Degradation of Carotenoids Under Low Level UV-B Radiation in the Filamentous Cyanobacterium Chlorogloeopsis fritschii PCC 6912
Source: Front Microbiol. 2020 Feb 12;11:163. doi: 10.3389/fmicb.2020.00163 (PMC7029182; doi:10.3389/fmicb.2020.00163)

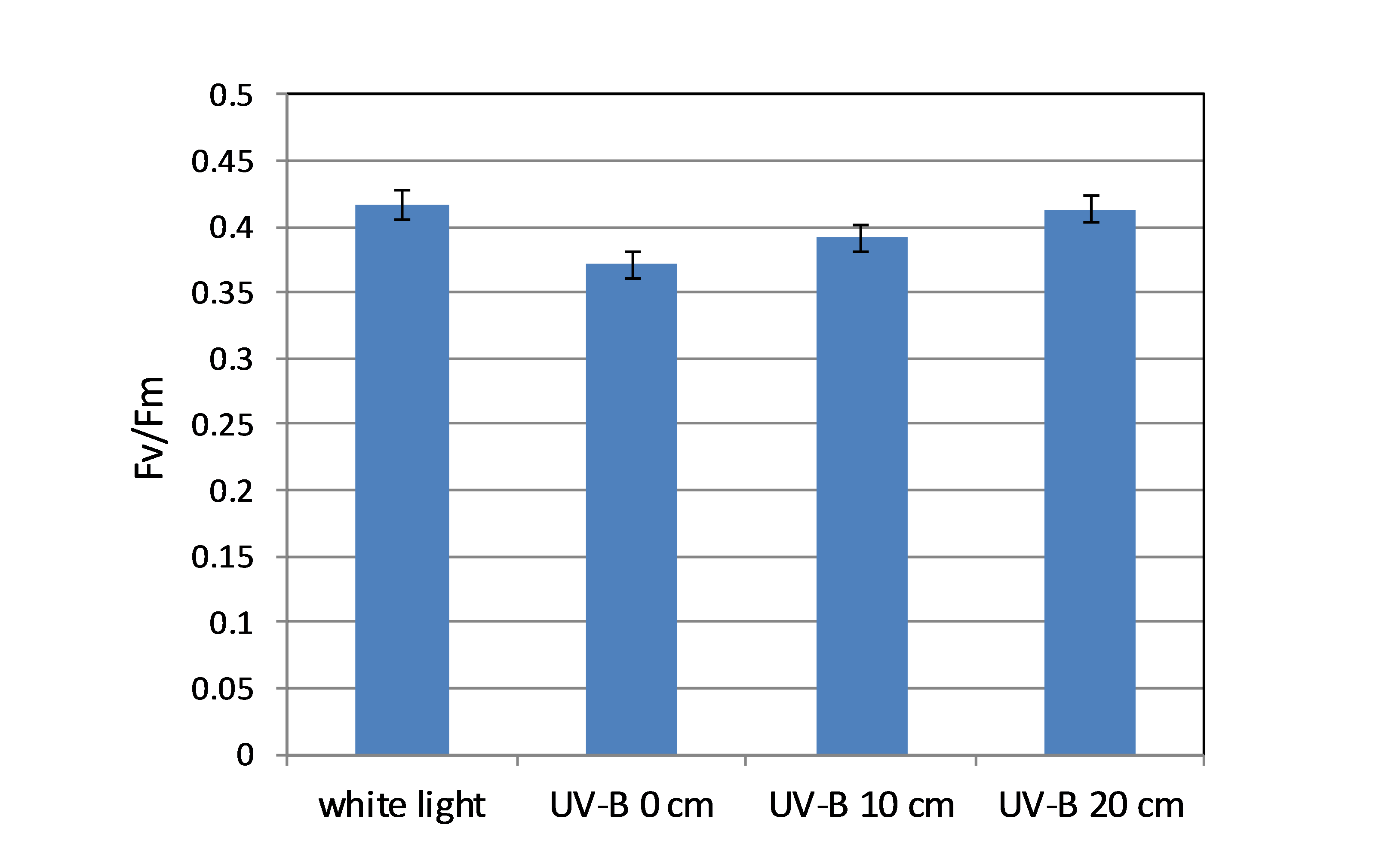

Supplement: FIGURE S1 — Prior experiment to determine level of UV-B at wavelength range 300–310 nm exposure that would not impact detrimentally on photosynthetic efficiency (Fv/Fm). White light, UV 0, 10, and 20 cm represent the control and the distance of the flasks containing C. fritschii PCC 6912 from the UVB source. Average of three replicates with standard deviation. From this, for the main experiment, flasks were placed 10 cm from UV-B tubes measured as supplying 3 μmol m–2 s–1. [file Image_1.tif]

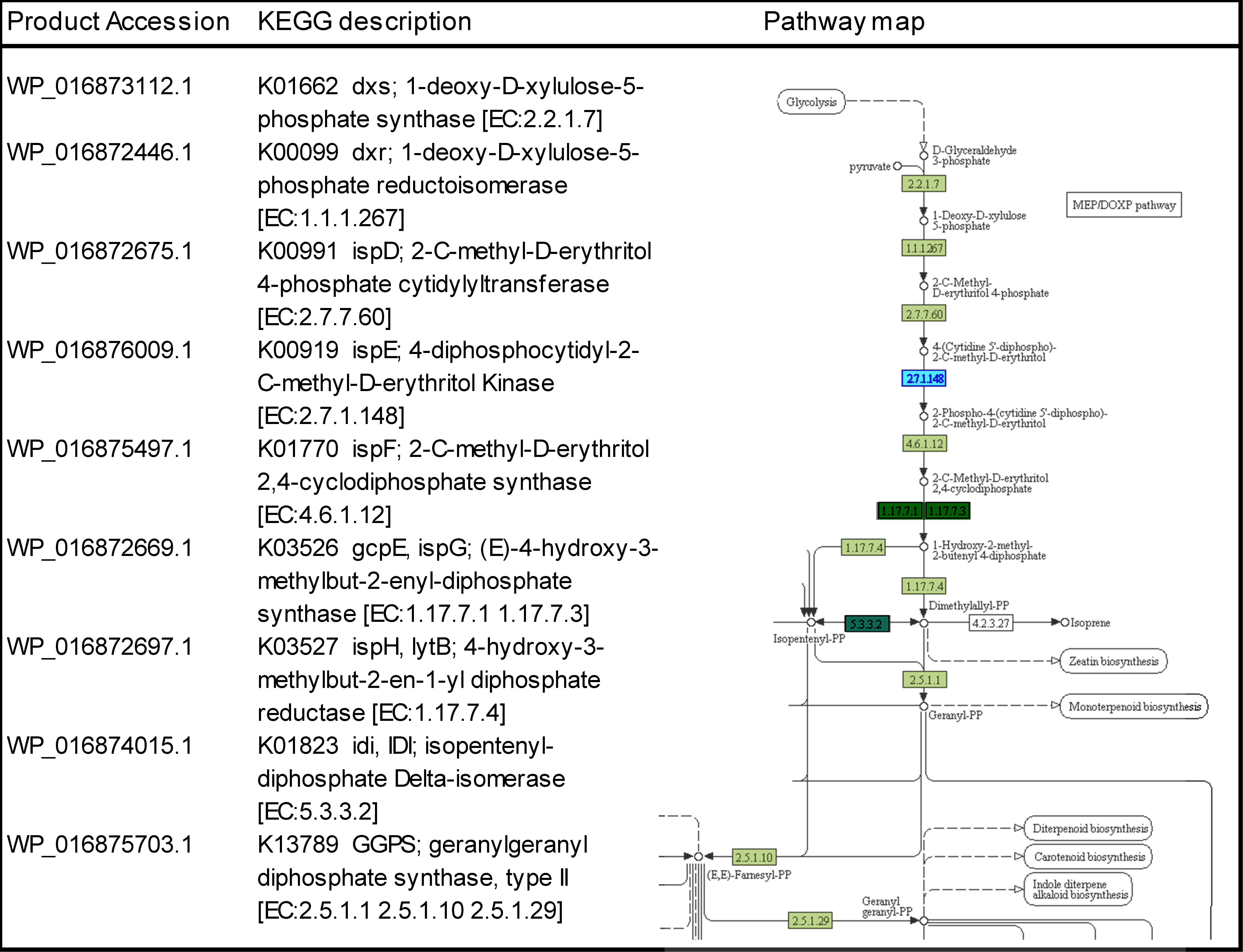

Supplement: FIGURE S3 — Gene regulation of terpenoid biosynthesis via the MEP/DOXP pathway to geranylgeranyl diphosphate in C. fritschii WP_016876009.1 was down-regulated (blue shading), other genes were identified but transcription was not observed (light green) or were not differentially regulated (dark green shading). [file Image_3.tif]
