# Supplementary material for: Synthesis, Regulation and Degradation of Carotenoids Under Low Level UV-B Radiation in the Filamentous Cyanobacterium Chlorogloeopsis fritschii PCC 6912
Source: Front Microbiol. 2020 Feb 12;11:163. doi: 10.3389/fmicb.2020.00163 (PMC7029182; doi:10.3389/fmicb.2020.00163)
Supplement: FIGURE S2 — HPLC chromatogram of C. fritschii PCC 6912. [file Image_2.pdf]

Supplementary Figure S2

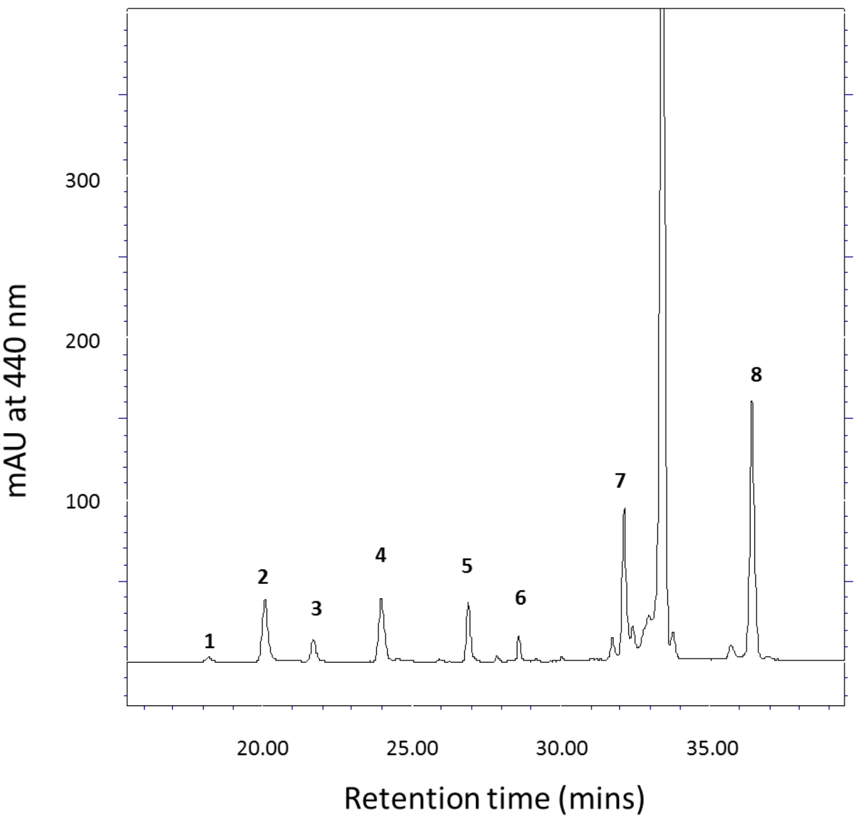

Figure S2: HPLC chromatogram of *C. fritschii PCC 6912.* Refer to Table S1 for details.
