# Supplementary material for: Synthesis, Regulation and Degradation of Carotenoids Under Low Level UV-B Radiation in the Filamentous Cyanobacterium Chlorogloeopsis fritschii PCC 6912
Source: Front Microbiol. 2020 Feb 12;11:163. doi: 10.3389/fmicb.2020.00163 (PMC7029182; doi:10.3389/fmicb.2020.00163)
Supplement: TABLE S1 — Retention time and UV/vis absorption maxima of main peaks detected during HPLC of C. fritschii PCC 6912. [file Table_1.docx]

Supplementary Table S1: Retention time and UV/vis absorption maxima of main peaks detected during HPLC of *C. fritschii PCC 6912:* Main ions (detected as protonated molecules) during LC/MS analysis, and assignment of key components. LC/MS^n^ was performed using an Agilent 1200 HPLC comprising a G1312A binary pump, a G1376B thermostated autosampler, a G1316A thermostated column compartment and a G1315B photodiode array detector coupled to an Agilent 6330 ion trap mass spectrometer via an atmospheric pressure chemical ionisation (APCI) source. MS settings were as follows: Ionisation mode: positive, drying temperature 350^o^C, vapouriser temperature 450^o^C, nebuliser pressure 60 PSI and drying gas flow rate 5 L/min. The scan range was *m/z* 200-1200. Components were assigned based on relative retention time, on-line UV/vis spectra, protonated molecule and fragmentation data (Airs et al. 2001).

| Retention time (min) | Peak number | UV/vis absorption bands (nm) | Main ions (LC/MS) | Assignment |
| --- | --- | --- | --- | --- |
| 18.18* | 1 | 449, 476, 508 | (747), 583, 565 | 2-hydroxy 2’-methylpentoside* |
| 20.07 | 2 | 430, 451, 479 | 601, 583, 565 | Nostoxanthin |
| 21.70 | 3 | 452, 476, 507 | (731), 567, 549 | Myxol 2’-methylpentoside |
| 23.98 | 4 | 430, 451, 479 | 585, 567 | Caloxanthin |
| 26.89 | 5 | 431, 453, 480 | 569, 551 | Zeaxanthin |
| 27.86* | * | 467 | 567, 549 | Unidentified |
| 28.58 | 6 | 475 | 565, 547 | Canthaxanthin |
| 32.12 | 7 | 461 | 551 | Echinenone |
| 33.40 |  | 430, 662 | 893 | Chlorophyll *a* |
| 36.41 | 8 | 453, 479 | 537 | β-carotene |

*Tentative assignment

Airs, R.L., Atkinson, J.E., and Keely, B.J. (2001). Development and application of a high resolution liquid chromatographic method for the analysis of complex pigment distributions. *Journal of Chromatography A* 917**,** 167-177.
