# Supplementary material for: Synthesis, Regulation and Degradation of Carotenoids Under Low Level UV-B Radiation in the Filamentous Cyanobacterium Chlorogloeopsis fritschii PCC 6912
Source: Front Microbiol. 2020 Feb 12;11:163. doi: 10.3389/fmicb.2020.00163 (PMC7029182; doi:10.3389/fmicb.2020.00163)
Supplement: FILE S1 — Promotor signatures of potential NtcA regulation of carotenoid transcription in C. fritschii PCC 6912. [file Data_Sheet_1.docx]

**Supplementary File S1: Promotor analysis for signatures of potential NtcA regulation of carotenoid transcription in *Chlorogloeopsis fritschii* PCC 6912**

NtcA is a global transcriptional regulator in cyanobacteria, noted for its role in nitrogen homeostasis. Its binding is enhanced by 2-oxoglutarate which acts as a signalling molecule for the cellular C to N balance (Tanigawa et al., 2002). NtcA has a wide role in the physiology of the cell including response to stress and the differentiation of heterocysts (Picossi et al., 2014).

Genes involved with keto carotenoid biosynthesis were found to be transcriptionally controlled by 2-oxoglutarate dependant binding of NtcA to the promoter regions in *Nostoc* PCC 7120, where the *crtW* gene and the *crtPB* operon are up-regulated in response to high light. (Sandmann et al., 2016)

Potential NtcA binding sites are recognizable in the promoter regions of *crtO*, *crtW* and the *crtPB* operon in *Nostoc* PCC 7120 (Sandmann et al., 2016) by their sequence motif. In nitrogen-regulated genes, the NtcA recognition and binding consensus is GTAN8TAC centred ~40 bp upstream from the transcription start site, with the GTN10AC subset being essential for binding. However, non-canonical binding sites can also be involved in NtcA-dependent regulation of transcription (Aldehni and Forchhammer, 2006).

Here, we analysed regions of the expressed genes for *crtO*, *crtW* and *crtPB* in *Chlorogloeopsis fritschii* PCC 6912 for putative NtcA binding regions in comparison with patterns of binding sites observed in *Nostoc* PCC 7120. Central positions of motifs are calculated relative to the transcription start site.

**Details of promotor features are given in Figure 1 below:-**

The expressed *C. fritschii* PCC 6912 *crtO* (WP_016878174.1) promotor has a GTN10AC motif at -55bp, corresponding to the putative NtcA binding motif identified in the Nostoc PCC 7120 all3744 gene at -50bp (GTAATTTCGTTTCAC). In *C. fritschii* PCC 6912 there is an additional upstream GTN10AC motif at -324 bp (GTCTGGGGCTATAC). Expression of *crtO* in *C. fritschii* PCC 6912 is not changed in response to UV, and is downregulated in *Nostoc* in high light.

The expressed *C. fritschii* PCC 6912 *crtW* (WP_016874500.1) upstream sequence has a non- canonical 13 bp GTN9AC motif at -65bp (GTGGTTAGTGAAC) which corresponds to the -64bp GTN9AC NtcA binding region in the *Nostoc* PCC 7120 *crtW* (alr3189) upstream sequence. Unlike the *Nostoc* sequence, there is a - 33bp GTN10AC motif (GTGTGAATTTAAAC) in the promotor region, and a third putative binding site - a GTN10AC motif at -108bp. The *Nostoc* sequence has a more distant GTN10AC motif at -327 bp. The transcription of *crtW* is upregulated in *C. fritschii* PCC 6912 under UV, and also in *Nostoc* in high light.

The upstream region of *C. fritschii* PCC 6912 phytoene desaturase *crtP* (WP_016875939.1) also has a 13 bp GTN9AC motif,( GTATAATGAGTAC) at the canonical position of -47bp, corresponding to the -47bp GTN9AC motif in the upstream region of *Nostoc* crtP (alr1832) (GTATAATGAGAAC). There are additional GTN10AC motifs in the *C. fritschii* PCC 6912 sequence at -150bp GTN10AC (GTTTGGGTGTCTAC) and -201bp GTN10AC (GTTTAAAATTCTAC) which are echoed by GTN11C motifs in *Nostoc* at -159 bp GTN11C (GTCCTTGNCGACTCC) and -202 bp GTN11C (GTTCACATCTGAGC). The *crtPB* operon is upregulated in *C. fritschii* PCC 6912 under UV, and in *Nostoc* in high light.

The conserved motifs illustrated here provide evidence for a functional role in the NtcA control of carotenoid transcription in *C. fritschii* PCC 6912, and provide the basis for further analytical investigation.

>crtO (WP_016878174.1) upstream sequence

CCAAGATTTTGGAGTTGCCCGCAGACTAAAA**GTCTGGGGCTATAC**AAACAAAGACTGTCTACGCAGTCTGATAACATTTCACCTCAATCACGATACACATATCATTATTGTAGAGACGCGATATATCGCGTCTCTACATGGTCTATATTTGTATCAGACTTTTTATGAAATGGTATTACATATTAATTATTTTTGCTCGAAAGCTTGAGACCATCAGCAGTTACCATCCCCCTACAGATTGATTGCAAACAACTGCTAAATCAAAGAAATGCTAAAGATGGGATTATAACTGGCGAAAGA**GTTATATTCTCCAC**CCTCTTTCTAACATCGGGAAAGATACACCCATTTCCCAAATTGCAACT

-55 bp GTN10AC GTTATATTCTCCAC

-324 bp GTN10AC GTCTGGGGCTATAC

>crtW (WP_016874500.1 ) upstream sequence

ACTCCGCGGCTCAAAAGCCTTACAGAGCAAGAAATTTTGCTTACACAGTCACCATCTATGCAAATATGTGTAATATTATTTAAGTAAACTTAATTTTTCTACATATTTGATTTAGAT**GTTACTTATAGCAC**AGCGCTGACCACGAACGGGGTTTTCCCTTAA**GTGGTTAGTGAAC**TTTTTAATGACCAAATT**GTGTGAATTTAAAC**AAAATTTTAGGCGGAGTAAAAGCCTG

-33bp GTN10AC GTGTGAATTTAAAC

-64bp GTN9AC GTGGTTAGTGAAC

-108bp GTN10AC GTTACTTATAGCAC

>crtPB (WP_016875939.1) upstream sequence

CAAATGTGGG**GTTTAAAATTCTAC**TAAAAGCAGTGCTTAACCATCGGGCTGACTCACCGCT**GTTTGGGTGTCTAC**AAAGGAGGAGAAGCGTCGCACGGCGAAGCTCTCTGCCCTCGGCTTTTTTACAATCATCTGTCATCGACTGCAAATGTAACGGTTTGCAAA**GTATAATGAGTAC**GGCAAAACCATTAAGAAATATTGAAGAGCAGTAAGGTTAA

-47bp GTN9AC GTATAATGAGTAC

-150bp GTN10AC GTTTGGGTGTCTAC

-201bp GTN10AC GTTTAAAATTCTAC

Supplementary File S1 Figure 1: NtcA binding sites in selected promoter regions of *C. fritschii* PCC 6912. Central positions of the 14bp motifs are given (-7bp from the 3’ end) calculated relative to the transcription start site. Putative Sigma70 promotor sequences predicted by BacPP software (de Avila e Silva et al., 2011) are shown in red (underlined) and putative NtcA binding sites are differentially coloured as indicated.

**References**

Aldehni, M.F., and Forchhammer, K. (2006). Analysis of a non-canonical NtcA-dependent promoter in Synechococcus elongatus and its regulation by NtcA and PII. *Arch Microbiol* 184**,** 378-386.

De Avila E Silva, S., Echeverrigaray, S., and Gerhardt, G.J.L. (2011). BacPP: Bacterial promoter prediction—A tool for accurate sigma-factor specific assignment in enterobacteria. *Journal of Theoretical Biology* 287**,** 92-99.

Picossi, S., Flores, E., and Herrero, A. (2014). ChIP analysis unravels an exceptionally wide distribution of DNA binding sites for the NtcA transcription factor in a heterocyst-forming cyanobacterium. *BMC Genomics* 15**,** 22.

Sandmann, G., Mautz, J., and Breitenbach, J. (2016). Control of light-dependent keto carotenoid biosynthesis in Nostoc 7120 by the transcription factor NtcA. *Z Naturforsch C* 71**,** 303-311.

Tanigawa, R., Shirokane, M., Maeda, S.-I., Omata, T., Tanaka, K., and Takahashi, H. (2002). Transcriptional activation of NtcA-dependent promoters of *Synechococcus* sp. PCC 7942 by 2-oxoglutarate *in vitro*. *Proceedings of the National Academy of Sciences* 99**,** 4251-4255.
